# Artificial Intelligence-Assisted Intravascular Imaging in Percutaneous Coronary Intervention: Current Status and Advances

**DOI:** 10.31083/RCM49881

**Published:** 2026-08-18

**Authors:** Zihan Li, Yuwei Wang, Ben Hu, Ziyang Ren, Jiawei Wu

**Affiliations:** ^1^Department of Cardiology, The First Affiliated Hospital of USTC, Division of Life Science and Medicine, University of Science and Technology of China, 230001 Hefei, Anhui, China; ^2^Anhui Medical University, 230000 Hefei, Anhui, China

**Keywords:** artificial intelligence, intravascular imaging, intravascular ultrasound, optical coherence tomography, percutaneous coronary intervention

## Abstract

Over the past two decades, intravascular imaging (IVI) has been widely integrated into clinical practice. Unlike traditional angiography, IVI uses an intracoronary imaging catheter to generate high-resolution cross-sectional images of diseased coronary arteries, thereby facilitating lesion characterization. These detailed images enable more accurate guidance of percutaneous coronary intervention (PCI). The clinical utility of IVI is demonstrated across four key stages: preoperative lesion assessment, intraoperative procedural guidance, postoperative complication detection, and evaluation of stent optimization. Despite these benefits, interventionalists continue to face challenges in consistently acquiring and interpreting high-quality IVI data. The emergence of artificial intelligence (AI) offers a promising solution by enabling precise image recognition and automated quantitative analysis, thereby supporting efficient clinical decision-making and broadening the scope of IVI applications. This review aims to summarize recent research progress in AI-assisted IVI across the PCI process and discuss future directions to improve the intelligence and efficiency of intravascular imaging.

## 1. Introduction

Percutaneous coronary intervention (PCI) has achieved rapid progress in recent years. Although coronary angiography remains the gold standard, novel techniques such as fractional flow reserve (FFR) and intravascular imaging (IVI) are playing increasingly vital roles in PCI, ushering coronary interventional medicine into a more precise era [1]. Relying exclusively on coronary angiography provides two-dimensional images of the coronary arteries, which are insufficient for a comprehensive assessment of plaque morphology. This limitation is particularly pronounced in vessels exhibiting positive remodeling or negative remodeling. In contrast, IVI can provide detailed cross-sectional data of the lesion, thus optimizing stent implantation and revascularization strategies while reducing the incidence of post-procedural adverse events. Over the years, numerous clinical studies have demonstrated the benefits of IVI-guided PCI [2,3,4,5,6,7,8,9] (Table 1). For instance, a randomized controlled trial focusing on patients with acute coronary syndrome and a subsequent network meta-analysis have further confirmed these findings [6,10]. Accordingly, the European Society of Cardiology for the Management of Chronic Coronary Syndromes now designates IVI-guided PCI for complex lesions as a Class Ia recommendation [11], underscoring its clinical significance.

**Table 1.** **Summary of randomized controlled trials on IVI-guided vs Angio-guided PCI for coronary artery disease**.

Sr. No.	Study name [Ref. #], year	Modality	Enrolled (IVI/Angio)	Follow-Up	Population characteristics	Primary endpoint (IVI group vs Angio group)
1.	IVUS-XPL [2], 2020	IVUS vs Angio	700/700	5 y	Patients with long coronary lesions (implanted stent length ≥28 mm)	MACE: 5.6% vs 10.7% (HR = 0.50; 95% CI: 0.34–0.75, *p* = 0.001)
2.	OCTOBER [3], 2023	OCT vs Angio	600/601	2 y	Patients with a clinical indication for PCI and a complex bifurcation lesion identified by means of coronary angiography	MACE: 10.1% vs 14.1% (HR = 0.70, 95% CI: 0.50–0.98, *p* = 0.035)
3.	ILUMIEN IV [4], 2023	OCT vs Angio	1233/1254	2 y	Patients with comorbid diabetes mellitus or complex coronary artery lesions	TVF: 7.4% vs 8.2% (HR = 0.90, 95% CI: 0.67–1.19, *p* = 0.45) The minimum stent area after PCI was 5.72 ± 2.04 mm^2^ in the OCT group and 5.36 ± 1.87 mm^2^ in the angiography group (mean difference, 0.36 mm^2^; 95% CI: 0.21 to 0.51; *p* < 0.001)
4.	RENOVATE-COMPLEX-PCI [5], 2023	IVUS/OCT vs Angio	1092/547	median follow-up time: 2.1 y	Patients with complex coronary-artery lesions (including bifurcation lesions, chronic total occlusion, unprotected left main coronary artery)	TVF: 7.7% vs 12.3% (HR = 0.64, 95% CI: 0.45–0.89, *p* = 0.008)
5.	IVUS-ACS [6], 2024	IVUS vs Angio	1753/1752	1 y	Patients with acute coronary syndrome	TVF: 4.0% vs 7.3% (HR = 0.55, 95% CI 0.41–0.74, *p* = 0.0001)
6.	OCCUPI [7], 2024	OCT vs angio	803/801	1 y	Patients who met the criterion of one or more complex lesions	MACE: 5% vs 7% (HR = 0.62, 95% CI 0.41 to 0.93, *p* = 0.023)
7.	ECLIPSE [8], 2025	IVUS/OCT vs angio	1246/759	1 y	Patients with severely calcified lesions (calcification length ≥15 mm)	TVF: 9.3% vs 13.2% (HR = 0.74, 95% CI: 0.56–0.97, *p* = 0.03)
8.	CALIPSO [9], 2025	OCT vs angio	65/69	/	Patients with stable moderate to severe calcified coronary lesions on coronary angiography scheduled for PCI	The final median (IQR) MSA: 6.5 [5.5–8.1] mm^2^ vs 5.0 [4.1–6.1] mm^2^; *p* < 0.001

Angio, angiography; CI, confidence interval; IVI, intravascular imaging; IVUS, intravascular ultrasound; IQR, interquartile range; HR, hazard ratio; MSA, minimum stent area; MACE, major adverse cardiac event; OCT, optical coherence tomography; PCI, percutaneous coronary intervention; TVF, target vessel failure; y, years.

Currently, the most commonly used IVI modalities in clinical practice include intravascular ultrasound (IVUS), optical coherence tomography (OCT), and near-infrared spectroscopy (NIRS). Given their unique imaging principles, each IVI modality has its own advantages and limitations in clinical application. The frequency range of commonly used IVUS transducers is 40–60 MHz. Based on ultrasound reflection imaging, this modality offers strong tissue penetration, is unaffected by blood interference and clearly displays the full thickness of the vessel wall. This has established IVUS as the gold standard for measuring the minimum lumen area and assessing plaque burden (PB). However, its relatively low-resolution limits its ability to characterize the fine microstructure of plaques. Although second-generation IVUS technologies (such as Virtual Histology IVUS and Integrated Backscatter IVUS) have attempted to address this limitation through tissue characterization [12,13,14,15], they still face challenges in accurately identifying vulnerable plaques. The integration of near-infrared spectroscopy with intravascular ultrasound (NIRS-IVUS) has partially addressed this limitation, enabling the accurate identification of lipid-rich vulnerable plaques [16], which have been closely associated with adverse cardiovascular events [17]. Waksman et al. [18] conducted the lipid-rich plaque (LRP) study, which further validated NIRS-IVUS as a novel risk-stratification tool in clinical practice.

OCT achieves imaging based on the principles of reflection and interference of near-infrared light, boasting higher spatial resolution (10–20 μm axial resolution) that can offer clear visualization of plaque microstructure. Because of this advantage, OCT holds an irreplaceable position in assessing fibrous cap thickness, detecting macrophage infiltration, and measuring dissection angles and lengths [19]. However, since light penetration into tissue is limited (approximately 1.0–2.5 mm), blood clearance by contrast flushing is required during OCT imaging. Accordingly, OCT remains slightly inferior to IVUS for visualizing the full thickness of the vessel wall.

The rapid evolution of artificial intelligence (AI), especially machine learning and deep learning (DL), has established a new technical approach for modern cardiovascular imaging. Machine learning, with its powerful computing and self-learning capabilities, aligns well with the modern medical demand for efficient and precise diagnosis. It has been widely explored for its role in assisting clinical decision-making, particularly in cardiovascular medicine. Machine learning mainly includes three major categories: supervised learning, unsupervised learning, and reinforcement learning. Supervised learning, which includes methods such as regularized regression, decision trees, and support vector machines, is commonly employed for risk prediction and classification [20]. In contrast, unsupervised learning focuses on exploring potential relationships between variables and serves as the theoretical foundation of DL [21]. Reinforcement learning integrates features of supervised and unsupervised learning, enabling iterative optimization of algorithmic performance through interaction with the environment. As an extension of machine learning, DL makes convolutional neural networks (CNN) as its core, being utilized to classify and segment images, which has been widely applied in clinical practice, such as computed tomography pulmonary nodule detection [22], the identification of atrial fibrillation in electrocardiograms [23] and computed tomography fractional flow reserve (CT-FFR) analysis of coronary computed tomography angiography [24]. Consequently, this review aimed to systematically examine advances in AI-assisted IVI and its potential future development directions.

## 2. AI-Assisted Intravascular Imaging for Preoperative Assessment

### 2.1 Accurate Segmentation and Quantification of Plaque Burden

Numerous prior studies have shown that IVUS exhibits high sensitivity in identifying vascular wall structures, detecting plaques, and quantifying PB [25]. Accurate assessment of PB can directly guide the selection of stent landing zones. The 2018 Expert Consensus on Intracoronary Imaging indicates that stent implantation in regions with a PB exceeding 50% is associated with an increased risk of restenosis [26]. Recent studies have demonstrated that AI can achieve high-precision segmentation of the lumen and external elastic membrane (EEM) in IVUS images, thereby identifying optimal landing zones through rapid, accurate determination of stenosis severity and delineation of plaque distribution. Blanco et al. [27] developed a fully automated segmentation model for lumen and vascular contours based on a multi-frame convolutional neural network (MFCNN) and a Gaussian process (GP) regressor, and its results demonstrated high concordance with expert human assessments. A Canadian study introduced a fully convolutional DL model based on a Dual Path U-Net and data augmentation strategies, demonstrating the AI segmentation model’s excellent generalization capability [28]. Hammouche et al. [29] developed an IVUS lumen segmentation algorithm based on a 3D adaptive spiral model. This approach achieves high segmentation accuracy (Dice index >94%) and fast computation (0.07 seconds per frame), while effectively handling complex coronary IVUS images with stenoses, artifacts, and other challenging features [29]. In addition, to address the issue that common artifacts in IVUS images (such as severe calcification and vessel bifurcation) compromise segmentation accuracy, a novel algorithm developed by a UK team has demonstrated precise segmentation in these challenging scenarios while effectively suppressing the artifacts [30]. In recent years, several research teams have developed a range of AI models for IVUS images acquired at different transducer frequencies, all achieving high detection accuracy and strong clinical applicability [31,32,33].

Compared with IVUS, OCT has traditionally been difficult to use to accurately assess PB due to its limited tissue penetration depth. However, the integration of AI is overcoming these anatomical limitations. Huang et al. [34] systematically validated the accuracy and feasibility of a DL-based OCT algorithm for the automated quantification of PB. Similarly, Chu et al. [35] developed a CNN-based model that achieved high diagnostic accuracy in the automated assessment of PB from OCT. Gao et al. [36] proposed the privileged modality distillation framework, which achieved exceptional accuracy (Dice index >95%) in dual-modality vascular boundary detection using IVUS and OCT. Collectively, these studies indicate that AI not only improves analytical efficiency but also delivers precise lesion characterization (Table 2, Ref. [27,28,29,30,31,32,33,34,35,36]), thereby providing reliable quantitative references for interventional cardiologists and facilitating precision cardiovascular medicine.

**Table 2.** **Summary of AI-based segmentation and quantification models for coronary IVUS and OCT images**.

Sr. No.	Author name [Ref. #], Year	Implemented technique	Transducer frequency (MHz)	Dataset	Evaluation metric	Technical advantages
**IVUS**						
1.	Yang et al. [28], 2019	Fully Convolutional Neural Network Dual Path U-Net	For dataset A- 40 MHz For dataset B- 20 MHz	Dataset A: 78 IVUS images Dataset B: 435 IVUS images from 10 patients	Dataset A: JI (lumen): 0.87 HD (lumen): 0.82 mm JI (media): 0.86 HD (media): 1.07 mm Dataset B: JI (lumen): 0.90 HD (lumen): 0.25 mm JI (media): 0.92 HD (media): 0.30 mm	1. The dual-path architecture is well-suited for small-sample training; 2. Artifact-specific augmentation enhances generalization capability; 3. The single-frame segmentation time is only 0.03 seconds; 4. Supports multi-frequency data of 20/40 MHz.
2.	Hammouche et al. [29], 2019	3D adaptive helix model	20 MHz	8918 IVUS images from 19 pullbacks	JI: 0.8855 ± 0.0509 DI: 0.9385 ± 0.0297 HD: 0.275 ± 0.139 mm Accuracy: 98.5%	1. No initialization close to the target is required, featuring a fully automated workflow; 2. Boasts robust 3D reconstruction capability.
3	Huang et al. [31], 2020	Select-P-MSER (MSER is the maximally stable extremal region)	20 MHz & 40 MHz	Dataset I Publicly available dataset A and dataset B Self-established and single- frame dataset: 300 IVUS images of 3 patients at 40 MHz	Dataset A: JI (Lumen): 0.77 ± 0.09 HD (Lumen): 1.21 ± 0.68 mm PAD (Lumen): 0.23 ± 0.18 JI (Media): 0.83 ± 0.08 HD (Media): 1.13 ± 0.69 mm PAD (Media): 0.11 ± 0.10 Dataset B: JI (Lumen): 0.89 ± 0.05 HD (Lumen): 0.28 ± 0.17 mm PAD (Lumen): 0.10 ± 0.08 JI (Media): 0.84 ± 0.11 HD (Media): 0.47 ± 0.31 mm PAD (Media): 0.13 ± 0.13	1. Strong adaptability across multiple frequencies (20/40 MHz); 2. Excellent resistance to artifacts (guidewire/shadow).
4	Ziemer et al. [32], 2020	MFCNN+Gaussian Process (GP) Regression	UR	23,970 IVUS images from 85 IVUS pullbacks of 52 patients	Relative distance error: 3.02 (2.25–3.95)% Maximum distance error: 10.3 (7.2–16.0)% Relative area error: 5.12 (2.15–9.00)%	Adaptable to large-scale IVUS image datasets from multiple patients.
5	Bajaj et al. [30], 2021	Pix2pix conditional Generative Adversarial Network (GAN).	50 MHz	26,211 IVUS images from 197 IVUS pullbacks of 65 patients	DI (Lumen): 0.96 ± 0.03 HD (Lumen): 0.19 ± 0.16 mm IoU (Lumen): 0.92 ± 0.05 DSC (Media): 0.98 ± 0.02 HD (Media): 0.15 ± 0.17 mm IoU (Media): 0.96 ± 0.03	Strong robustness in complex scenarios (calcification/collateral vessels).
6	Zhu et al. [33], 2022	IVUS-U-Net++ (Deep learning with feature pyramid network)	40 MHz	1746 IVUS images from 18 patients	JI (Lumen): 0.9080 ± 0.0321 HD (Lumen): 0.1484 ± 0.1584 mm JI (Media): 0.9199 ± 0.0370 HD (Media): 0.1781 ± 0.1906 mm	1. The multiscale feature representation and fusion capability have been enhanced to a certain extent; 2. It integrates multi-scale features, effectively addressing the insufficient feature fusion of traditional U-Net-like models.
7	Blanco et al. [27], 2022	MFCNN+GP Regression	20 MHz	13,435 IVUS images from 160 IVUS pullbacks of 63 patients	JI (Lumen): 0.913, IQR: (0.882, 0.935) JI: 0.940, IQR: (0.917, 0.957) LA: r = 0.98 (*p* < 0.01) PB: r = 0.95 (*p* < 0.01)	1. Utilizes inter-frame adjacent information, resulting in strong segmentation continuity; 2. GP regression filters noise, yielding smooth contours.
**OCT**						
8	Gao et al. [36], 2020	Privileged Modality Distillation (PMD)	20 MHz	2018 IVUS images and 2018 OCT images	For IVUS: DI (Lumen): 0.95 JI (Lumen): 0.92 HD (Lumen): 0.22 mm ± 0.15; DI (Media): 0.96 JI (Media): 0.92 HD (Media): 0.29 mm ± 0.22 mm For OCT Lumen: DI: 0.97 JI: 0.95 HD: 0.16 mm ± 0.20 mm	Excellent clinical compatibility: compatible with both IVUS and OCT imaging modalities.
9	Chu et al. [35], 2021	U-shaped encoder-decoder with pseudo-3D input and hybrid loss	Frequency-domain OCT systems used, not IVUS	Internal Dataset: 509 OCT pullbacks (391 patients); 10,517 training frames and 1156 testing frames. External Validation Dataset: 300 OCT frames from 30 patients.	The PB assessed by the model correlated very well with the ground truth (R^2^ = 0.98, *p* < 0.001), with a mean difference of 0.35 ± 2.2% and an ICCa of 0.99 (95% CI: 0.98–0.99). Average speed of 0.07 ± 0.01 seconds per cross-section	The model uses pseudo-3D input—stacked consecutive OCT cross-sections as separate color channels—to integrate spatial information which can reduce feature loss during downsampling.
10	Huang et al. [34], 2023	Deep learning (U-shaped encoder-decoder CNN applied to OCT)	20 MHz (for validation IVUS)	Validation: 64 paired OCT and IVUS cross-sections from 15 patients. DL Training: 11,673 OCT cross-sections.	PB Concordance: ICC = 0.81 PB Difference: –3.53 ± 6.17% Diagnostic Accuracy (PB >65%): 92% Lumen Area: ICC = 0.85 Vessel Area: ICC = 0.72	Adapts to the low penetration depth of OCT, breaking through the limitation that traditional OCT cannot accurately quantify PB.

CI, confidence interval; CNN, convolutional neural network; DI, dice index; DL, deep learning; DSC, dice similarity coefficient; GAN, generative adversarial network; HD, hausdorff distance; ICC, intraclass correlation coefficient; ICCa, adjusted intraclass correlation coefficient; IVUS, intravascular ultrasound; IQR, interquartile range; JI, jaccard index; IoU, intersection over union; LA, lumen area; MFCNN, multi-frame convolutional neural network; MSER, maximally stable extremal region; OCT, optical coherence tomography; PAD, percentage of area difference; PB, plaque burden; PMD, privileged modality distillation; UR, unreported.

### 2.2 Plaque Characterization

In clinical practice, plaque phenotype is directly linked to the severity of coronary lesions. Atherosclerotic lesions characterized by a large lipid-rich core and a thin fibrous cap are termed thin-cap fibroatheroma (TCFA). These high-risk lesions are closely associated with the occurrence of acute coronary syndrome and often indicate a poor clinical prognosis [37,38]. However, severe calcification frequently results in suboptimal stent expansion, thereby increasing the risk of adverse perioperative events, such as in-stent thrombosis and restenosis [39,40]. Thus, accurate characterization of plaque features is critical, and AI offers a more efficient strategy for the precise identification and quantification of coronary atherosclerotic plaques.

OCT provides high-resolution imaging information on plaque characteristics, and AI technology has substantially improved the efficiency and accuracy of OCT for coronary plaque detection and analysis. A South Korean research team randomly divided 602 coronary lesions from 602 patients with angina into a training set and a test set at a 4:1 ratio. They developed a DenseNet model to automatically detect TCFA, which achieved an overall accuracy of 92.8% on the test set and showed a significant correlation with expert identification (*r* = 0.87, *p* < 0.001) [41]. Chu et al. [35] developed and validated a deep CNN-based AI model that not only achieved high accuracy in automatically assessing PB but also enabled automatic identification of plaque characteristics. The model achieved its highest diagnostic accuracy in the classification of fibrous plaques (accuracy 97.6%, 95% confidence interval [CI]: 93.4–99.3%), followed by lipidic plaque (90.5% [95% CI: 85.2–94.1%]) and calcifications (88.5% [95% CI: 82.4–92.7%]) [35]. Building on this work, the investigators further developed another model, dual-coordinate cross-attention transformer (DCCAT), designed to identify thrombus location and area in OCT images. On an external test set, it achieved a Dice similarity coefficient (DSC) of 0.706 for thrombus segmentation, significantly outperforming the traditional CNN (DSC: 0.656) and the transformer-based model (DSC: 0.584) [42]. Abdolmanafi et al. [43] developed an AI model for multi-type atherosclerotic lesion classification including thrombus based on the ResNet architecture, achieving an accuracy of up to 98% for thrombus, comparable to that of human experts. Moreover, in recent years, several studies have focused on developing AI models based on OCT images, enabling automated measurement of fibrous cap thickness and discrimination of plaque tissue components [44,45,46]. OCT offers a distinct advantage in identifying calcified plaques. Avital et al. [47] developed a DL algorithm based on the U-Net that achieved automated segmentation of coronary calcifications in OCT images, with an accuracy of 0.9903 ± 0.009. Meanwhile, American research teams created a segmentation network-derived model that automatically segments and quantifies calcification features, achieving a calcification sensitivity of 0.85 ± 0.04. The model automatically quantifies lumen area, calcification arc, thickness, and depth, with a segmentation time of less than one second per frame, offering an automated solution for planning pretreatment strategies for calcified lesions prior to PCI [48].

Compared with OCT, identifying plaque tissue components using IVUS is more challenging. The integration of AI technology has effectively addressed this difficulty, driving the upgrade of IVUS image analysis toward more intelligent and automated solutions. Recent advancements in AI have facilitated the high-fidelity classification of plaque morphology, which was previously a manual and subjective process [49]. Bae et al. [50] constructed a supervised machine learning model based on 41,101 co-registered IVUS-OCT frames from 517 patients. Among the models evaluated, the artificial neural network achieved the best performance in predicting TCFA as defined by OCT, with an accuracy of 82% (area under the curve [AUC] = 0.82) in the test set [50]. Masuda et al. [51] established a 7-layer U-Net++ DL model using integrated backscatter IVUS as the reference standard. This model enabled automated segmentation of the lumen and vessel wall on conventional IVUS images, as well as accurate quantification of lipid, fibrous, dense fibrous, and calcified tissues, with superior performance in lipid identification. The model predicted LRP with a sensitivity of 62% and a specificity of 94% [51]. Collectively, these advancements have significantly expanded the clinical utility of IVUS, enabling more robust and objective qualitative assessment of atherosclerotic lesions.

### 2.3 New Dimension in Functional Assessment

In current clinical practice, along with the advancement of high-resolution imaging techniques, the functional index, FFR, is also widely used in PCI, which can more accurately assess whether vascular stenosis truly causes myocardial ischemia from a functional perspective [52,53]. Although significant efforts have been undertaken to explore the association between vascular morphological characteristics and functional parameters over the past few years, the findings have not been entirely satisfactory [54,55]. With the rise of AI technology, its powerful computing and feature-extraction capabilities have opened new possibilities for overcoming this limitation. For example, a functional assessment method based on coronary CT-FFR stands as a landmark success of AI in the functional domain [56,57].

On this basis, more research teams have sought to combine IVI with FFR to achieve a deeper integration of morphological and functional assessments. In a study of 1328 coronary lesions, researchers found that an IVUS-based machine-learning model could accurately identify lesions that cause myocardial ischemia. By learning the relationships between numerous imaging features and FFR results, the model ultimately achieved a diagnostic accuracy exceeding 83% [58]. Another study by Cha et al. [59] demonstrated that an OCT-based AI model can also predict FFR results with relatively high accuracy. These models enable rapid localization of true ischemic culprit lesions in complex multivessel disease, thereby not only preventing unnecessary stent placement but also facilitating functionally successful interventions by predicting post-PCI FFR.

## 3. AI-Assisted Optimization of the PCI Procedure and Device Selection

The limitation of traditional coronary angiography has been addressed by the development and application of IVI in recent years. It has transformed coronary imaging from mere luminal morphological observation to detailed analysis of three-dimensional vascular structures. This can guide interventional procedures more efficiently and reduce adverse events following stent implantation [25]. Several randomized controlled trials have confirmed its clinical value. For example, the ILUMIEN III study enrolled 450 patients with coronary artery lesions across 29 hospitals in 8 countries, who were randomly assigned in a 1:1:1 ratio to undergo PCI guided by OCT, IVUS or angiography. The results demonstrated that compared with angiography-guided PCI alone, OCT-guided PCI significantly improved stent expansion (minimum stent expansion rate: 87.6% vs. 82.9%, *p* = 0.02) [60]. Based on precise pre-procedural measurements of lumen and vessel dimensions, quantitative analysis of PB, and comprehensive assessment of lesion characteristics, interventionalists can select more appropriate and personalized stent implantation strategies. Overall, AI-assisted IVI significantly enhances the accuracy of pre-operative lesion assessment, thereby providing stronger support for interventional planning.

However, in current clinical practice, operators mainly rely on visual assessment and manual measurements from angiography and IVI during PCI procedures, which are subjective, operator-dependent, time-consuming, and poorly replicable. The clinical adoption of IVI is currently hindered by the need for specialized expertise and a widespread lack of systematic training among clinicians [61]. This indicates that standardized training and intelligent assistive tools are needed in the future to improve the clinical, diagnostic and operational performance.

AI has been integrated across various medical sectors, demonstrating considerable potential to assist clinicians in formulating interventional strategies and selecting device sizes. By facilitating the automated identification and quantitative analysis of IVI features, AI provides the foundational technical support for precise preoperative planning. Existing studies have developed automated IVUS image segmentation technology based on DL methods that can accurately quantify lumen dimensions and provide objective evidence for device selection [28,62]. Matsumura et al. [63] developed a machine learning model, which achieved high accuracy in automatically segmenting coronary vessel structures, quantifying lumen size, and predicting appropriate balloon dimensions in their recent investigation. Furthermore, the AI-integrated IVUS system AVVIGO, developed by Boston Scientific, has demonstrated excellent performance in clinical validation. A recent study has already indicated that the system exhibits a high degree of concordance with human experts in selecting stent sizes and significantly enhances procedural efficiency [64].

More promisingly, many research teams are exploring the use of AI to predict outcomes in interventional procedures. A South Korean research team analyzed 618 lesions and developed an innovative DL model that can accurately predict post-stent-implantation expansion based solely on pre-procedural IVUS imaging. It can achieve a prediction accuracy of up to 94% [65]. The application of this model helps interventionalists identify high-risk lesions before the operation that are associated with inadequate stent expansion, thereby guiding personalized lesion preparation and device selection [65].

Iatrogenic vessel wall disruption, manifesting as dissections or hematomas, frequently complicates PCI procedures within the dilated or adjacent stent segments. With its superior tissue penetration, IVUS can clearly display the extent and depth of dissections and hematomas [66]. In contrast, with its higher spatial resolution, OCT can provide a detailed assessment of microscopic features, including dissection morphology, flap angle, extent, and intimal flap thickness [67]. According to the dissection information provided by IVI, interventionalists can select appropriately sized, length-matched stents to ensure complete coverage of the dissection entry and exit points, thereby significantly reducing the risk of postoperative restenosis or in-stent thrombosis. However, research on AI-assisted IVI that automatically identifies dissections and hematomas remains relatively limited. This area holds substantial potential and is expected to emerge as a primary focus of future AI-assisted IVI investigations.

## 4. AI-Assisted Identification of Post-PCI Complications

As mentioned above, IVI can assist in determining the need for intervention, optimizing stent size and length selection, and guiding optimal stent landing. It also plays a critical role in post-procedural assessment [68]. IVI can identify various complications after stent implantation, such as edge dissection, stent malapposition, tissue prolapse, and insufficient residual lumen area [69,70]. Thus, operators can use information provided by IVI to determine the need for additional intervention. Moreover, analysis of postoperative images can reveal potential causes of poor outcomes, such as stent fracture or in-stent restenosis [71,72]. In the field of automated stent imaging analysis, based on a large-scale dataset (1576 IVUS pullbacks) and an independent validation set, Nishi et al. [73] innovatively proposed a DL model capable of automatically segmenting the stent region and accurately quantifying stent dimensions. This work provides foundational support for the future quantitative analysis of inadequate stent expansion. However, the model exhibits limited ability to automatically identify major complications such as dissections, thrombi, tissue prolapse, and stent malapposition. This is still an important challenge that needs to be overcome in the future. Concurrently, other research teams have developed various DL models for postoperative IVUS images. The DL algorithm proposed by Wissel et al. [74] can efficiently and accurately segment the stent area in IVUS pullback image sequences, further verifying the potential of AI for post-procedural IVUS evaluation.

In contrast, OCT relies on its high spatial resolution, which enables clearer visualization of subtle features, such as stent strut apposition, tissue coverage, and stent strut distribution [75]. Wang et al. [76] proposed an innovative algorithmic framework that automates 3D detection of the stent mesh structure in OCT, reducing analysis time from several hours to approximately 2 minutes. This lays an important foundation for the intelligent quantitative analysis of OCT. Concurrently, the feasibility of automated OCT-based stent analysis has been independently validated by Xu et al. [77], Tsantis et al. [78], and Lu et al. [79]. In recent years, a team of Chinese scholars, based on 25,203 OCT images, developed a DL model, which can accurately differentiate stents based on tissue coverage thickness and was systematically validated for its applicability and robustness in vessels with multiple stents [80].

## 5. Comparison and Perspectives of Diverse AI Approaches

Current AI models for coronary intravascular image analysis can be compared along two dimensions: network architecture type and learning paradigm. Their performance characteristics, strengths, and limitations are closely tied to clinical applicability.

CNN-based models, including U-Net and its variants, MFCNN, and others, are currently the mainstream choice for IVI analysis. Their main advantage is the efficient extraction of local spatial features, which helps capture the morphology of structures such as the lumen and plaque. Through training, they can accurately segment the lumen and media, as demonstrated by Ziemer et al. [32], Hammouche et al. [29], and Gao et al. [36], among others. However, traditional CNNs are limited by their weak ability to capture long-range spatial dependencies, and their performance tends to degrade when dealing with complex lesions such as tortuous and bifurcated vessels. Moreover, some models, such as MFCNN, have high requirements for input sequence continuity and data quality. In recent years, compared with traditional CNNs, the emerging Transformer architecture has been shown to capture long-term dependencies [81], and recent studies have integrated it into medical imaging tasks to further improve AI model performance [82]. However, Transformer relies on large annotated datasets, incurs high computational cost and is vulnerable to artifacts such as guidewire shadows. As a result, its accuracy is slightly lower than that of optimized CNN variants.

In terms of learning methods, most AI models developed so far are based on supervised learning, which relies on large amounts of manually annotated “image-ground truth” data. Supervised learning, however, incurs extremely high annotation costs, and its generalizability is greatly affected by the distribution of annotated data. Semi-supervised learning addresses data insufficiency through limited annotation but lacks the precision of supervised models, rendering it less effective for plaque characterization than for coarse lumen segmentation.

While CNNs remain the standard for IVI analysis, Transformer architectures are increasingly employed for complex spatial tasks like bifurcation evaluation. Besides, weakly supervised learning is pivotal for optimizing large-scale, multi-center datasets by minimizing annotation costs and enhancing clinical scalability.

## 6. Opportunities and Challenges

Driven by rapid progress in AI, multiple AI-integrated IVI systems, including AVVIGO+ (Version 1.0, Boston Scientific Corporation, Marlborough, Massachusetts, USA), Ultreon OCT 2.0 (Version 2.0, Abbott, Abbott Park, Illinois, USA), and Gentuity HF-OCT (Gentuity, LLC, Sudbury, Massachusetts, USA), have emerged in recent years. These systems have been applied in the current catheterization laboratory, playing significant roles in pre-procedural assessment, intra-operative guidance, and post-procedural analysis of PCI [83]. Although such AI tools exhibit significant advantages in image processing and analysis efficiency, studies have shown that their measurements of lumen, vessel, and stent region dimensions are more conservative than those of human experts, with a potential tendency toward systematic underestimation [84]. Therefore, whether these AI-integrated IVI systems are feasible and reliable in clinical settings requires further validation through additional prospective studies.

Current evidence confirms that AI-integrated IVI optimizes the full spectrum of the PCI continuum, from initial lesion characterization to real-time guidance and long-term complication monitoring. Nevertheless, although most developed AI models have demonstrated good performance on validation or external test sets, their practical value in real clinical settings remains to be further confirmed. Nowadays, there is inadequate evidence to confirm that these models based on AI can significantly enhance procedural efficiency, improve clinical outcomes, or reduce the incidence of adverse events in actual PCI practice. Therefore, large-scale, multicenter randomized controlled trials are needed to systematically evaluate the real-world benefits of AI-assisted IVI in clinical practice. In addition, the diversity of sources for clinical intracoronary imaging devices leads to significant variations in imaging parameters and stored data formats among devices from different manufacturers. To address this, efforts should focus on advancing multi-center, multi-device data collaboration. Standardized data preprocessing, including unifying image resolution and calibrating the grayscale distribution, can enhance the model’s adaptability across diverse devices. Furthermore, for AI to be adopted in clinical practice, it requires not only higher efficiency and real-time, lag-free analysis but also standardized workflows, improved social regulatory mechanisms, well-defined data access permissions, and multidisciplinary collaboration. A study in an emergency setting reported that Picture archiving and communication system improves the accuracy of image interpretation [85]. This finding underscores the critical role of systematic infrastructure development. Therefore, better infrastructure could help promote wider acceptance of AI in coronary intervention.

Notably, AI research in IVI is gradually expanding to include more complex lesion types. More research teams are attempting to develop models capable of identifying and quantifying complex structural features, such as automatically detecting the presence and angle of dissections, and even exploring the feasibility of using AI to guide wire crossing paths in chronic total occlusion lesions. These pioneering explorations have opened new directions for AI-assisted precise interventional therapy and have also provided important support for developing intelligent and individualized interventional strategies in the future.

## 7. Conclusion

With the accumulation of clinical evidence, the application of IVI in PCI is increasingly recognized for improving long-term patient prognosis. With its powerful computing power, AI has been integrated into clinical practice, demonstrating remarkable efficiency and accuracy, and aligning perfectly with the evolving trend of precision medicine. Integrating AI with IVI can achieve more precise image recognition and quantitative analysis, while expanding the clinical application scope of IVI. Currently, multiple research groups are actively developing AI models for complex imaging analysis in IVI, while IVI systems integrated with AI have already entered clinical practice. Future large-scale prospective studies are expected to refine PCI procedures, offering renewed promise for patients with coronary artery disease.

## Abbreviations

AI, artificial intelligence; CNN, convolutional neural networks; IVI, intravascular imaging; IVUS, intravascular ultrasound; EEM, external elastic membrane; FFR, fractional flow reserve; NIRS, near-infrared spectroscopy; OCT, optical coherence tomography; PCI, percutaneous coronary intervention.

## References

[1] Groves EM, Seto AH, Kern MJ (2016). Invasive Testing for Coronary Artery Disease: FFR, IVUS, OCT, NIRS. Heart Failure Clinics.

[2] Hong SJ, Mintz GS, Ahn CM, Kim JS, Kim BK, Ko YG (2020). Effect of Intravascular Ultrasound-Guided Drug-Eluting Stent Implantation: 5-Year Follow-Up of the IVUS-XPL Randomized Trial. JACC. Cardiovascular Interventions.

[3] Holm NR, Andreasen LD, Neghabat O, Laanmets P, Kumsars I, Bennett J (2023). OCT or Angiography Guidance for PCI in Complex Bifurcation Lesions. The New England Journal of Medicine.

[4] Ali ZA, Landmesser U, Maehara A, Matsumura M, Shlofmitz RA, Guagliumi G (2023). Optical Coherence Tomography-Guided versus Angiography-Guided PCI. The New England Journal of Medicine.

[5] Lee JM, Choi KH, Song YB, Lee JY, Lee SJ, Lee SY (2023). Intravascular Imaging-Guided or Angiography-Guided Complex PCI. The New England Journal of Medicine.

[6] Li X, Ge Z, Kan J, Anjum M, Xie P, Chen X (2024). Intravascular ultrasound-guided versus angiography-guided percutaneous coronary intervention in acute coronary syndromes (IVUS-ACS): a two-stage, multicentre, randomised trial. Lancet (London, England).

[7] Hong SJ, Lee SJ, Lee SH, Lee JY, Cho DK, Kim JW (2024). Optical coherence tomography-guided versus angiography-guided percutaneous coronary intervention for patients with complex lesions (OCCUPI): an investigator-initiated, multicentre, randomised, open-label, superiority trial in South Korea. Lancet (London, England).

[8] Stone GW, Genereux P, Maehara A, Lewis BE, Shlofmitz RA, Dohad S (2025). Intravascular Imaging vs Angiography Guidance for PCI of Severely Calcified Lesions: The ECLIPSE Trial. JACC. Cardiovascular Interventions.

[9] Amabile N, Rangé G, Landolff Q, Bressollette E, Meneveau N, Lattuca B (2025). OCT vs Angiography for Guidance of Percutaneous Coronary Intervention of Calcified Lesions: The CALIPSO Randomized Clinical Trial. JAMA Cardiology.

[10] Stone GW, Christiansen EH, Ali ZA, Andreasen LN, Maehara A, Ahmad Y (2024). Intravascular imaging-guided coronary drug-eluting stent implantation: an updated network meta-analysis. Lancet (London, England).

[11] Vrints C, Andreotti F, Koskinas KC, Rossello X, Adamo M, Ainslie J (2024). 2024 ESC Guidelines for the management of chronic coronary syndromes. European Heart Journal.

[12] Diethrich EB, Pauliina Margolis M, Reid DB, Burke A, Ramaiah V, Rodriguez-Lopez JA (2007). Virtual histology intravascular ultrasound assessment of carotid artery disease: the Carotid Artery Plaque Virtual Histology Evaluation (CAPITAL) study. Journal of Endovascular Therapy : an Official Journal of the International Society of Endovascular Specialists.

[13] Sangiorgi G, Bedogni F, Sganzerla P, Binetti G, Inglese L, Musialek P (2013). The Virtual histology In CaroTids Observational RegistrY (VICTORY) study: a European prospective registry to assess the feasibility and safety of intravascular ultrasound and virtual histology during carotid interventions. International Journal of Cardiology.

[14] Kawasaki M, Hattori A, Ishihara Y, Okubo M, Nishigaki K, Takemura G (2010). Tissue characterization of coronary plaques and assessment of thickness of fibrous cap using integrated backscatter intravascular ultrasound. Comparison with histology and optical coherence tomography. Circulation Journal : Official Journal of the Japanese Circulation Society.

[15] Kawasaki M, Bouma BE, Bressner J, Houser SL, Nadkarni SK, MacNeill BD (2006). Diagnostic accuracy of optical coherence tomography and integrated backscatter intravascular ultrasound images for tissue characterization of human coronary plaques. Journal of the American College of Cardiology.

[16] Johnson TW, Räber L, di Mario C, Bourantas C, Jia H, Mattesini A (2019). Clinical use of intracoronary imaging. Part 2: acute coronary syndromes, ambiguous coronary angiography findings, and guiding interventional decision-making: an expert consensus document of the European Association of Percutaneous Cardiovascular Interventions. European Heart Journal.

[17] Cheng JM, Garcia-Garcia HM, de Boer SPM, Kardys I, Heo JH, Akkerhuis KM (2014). In vivo detection of high-risk coronary plaques by radiofrequency intravascular ultrasound and cardiovascular outcome: results of the ATHEROREMO-IVUS study. European Heart Journal.

[18] Waksman R, Di Mario C, Torguson R, Ali ZA, Singh V, Skinner WH (2019). Identification of patients and plaques vulnerable to future coronary events with near-infrared spectroscopy intravascular ultrasound imaging: a prospective, cohort study. Lancet (London, England).

[19] Mintz GS, Guagliumi G (2017). Intravascular imaging in coronary artery disease. Lancet (London, England).

[20] Johnson KW, Torres Soto J, Glicksberg BS, Shameer K, Miotto R, Ali M (2018). Artificial Intelligence in Cardiology. Journal of the American College of Cardiology.

[21] Krittanawong C, Zhang H, Wang Z, Aydar M, Kitai T (2017). Artificial Intelligence in Precision Cardiovascular Medicine. Journal of the American College of Cardiology.

[22] Winkels M, Cohen TS (2019). Pulmonary nodule detection in CT scans with equivariant CNNs. Medical Image Analysis.

[23] Attia ZI, Noseworthy PA, Lopez-Jimenez F, Asirvatham SJ, Deshmukh AJ, Gersh BJ (2019). An artificial intelligence-enabled ECG algorithm for the identification of patients with atrial fibrillation during sinus rhythm: a retrospective analysis of outcome prediction. Lancet (London, England).

[24] Kumamaru KK, Fujimoto S, Otsuka Y, Kawasaki T, Kawaguchi Y, Kato E (2020). Diagnostic accuracy of 3D deep-learning-based fully automated estimation of patient-level minimum fractional flow reserve from coronary computed tomography angiography. European Heart Journal. Cardiovascular Imaging.

[25] Truesdell AG, Alasnag MA, Kaul P, Rab ST, Riley RF, Young MN (2023). Intravascular Imaging During Percutaneous Coronary Intervention: JACC State-of-the-Art Review. Journal of the American College of Cardiology.

[26] Räber L, Mintz GS, Koskinas KC, Johnson TW, Holm NR, Onuma Y (2018). Clinical use of intracoronary imaging. Part 1: guidance and optimization of coronary interventions. An expert consensus document of the European Association of Percutaneous Cardiovascular Interventions. European Heart Journal.

[27] Blanco PJ, Ziemer PGP, Bulant CA, Ueki Y, Bass R, Räber L (2022). Fully automated lumen and vessel contour segmentation in intravascular ultrasound datasets. Medical Image Analysis.

[28] Yang J, Faraji M, Basu A (2019). Robust segmentation of arterial walls in intravascular ultrasound images using Dual Path U-Net. Ultrasonics.

[29] Hammouche A, Cloutier G, Tardif JC, Hammouche K, Meunier J (2019). Automatic IVUS lumen segmentation using a 3D adaptive helix model. Computers in Biology and Medicine.

[30] Bajaj R, Huang X, Kilic Y, Ramasamy A, Jain A, Ozkor M (2021). Advanced deep learning methodology for accurate, real-time segmentation of high-resolution intravascular ultrasound images. International Journal of Cardiology.

[31] Huang Y, Yan W, Xia M, Guo Y, Zhou G, Wang Y (2020). Vessel membrane segmentation and calcification location in intravascular ultrasound images using a region detector and an effective selection strategy. Computer Methods and Programs in Biomedicine.

[32] Ziemer PGP, Bulant CA, Orlando JI, Maso Talou GD, Álvarez LAM, Guedes Bezerra C (2020). Automated lumen segmentation using multi-frame convolutional neural networks in intravascular ultrasound datasets. European Heart Journal. Digital Health.

[33] Zhu F, Gao Z, Zhao C, Zhu H, Nan J, Tian Y (2022). A Deep Learning-based Method to Extract Lumen and Media-Adventitia in Intravascular Ultrasound Images. Ultrasonic Imaging.

[34] Huang J, Tu S, Masuda S, Ninomiya K, Dijkstra J, Chu M (2023). Plaque burden estimated from optical coherence tomography with deep learning: In vivo validation using co-registered intravascular ultrasound. Catheterization and Cardiovascular Interventions : Official Journal of the Society for Cardiac Angiography & Interventions.

[35] Chu M, Jia H, Gutiérrez-Chico JL, Maehara A, Ali ZA, Zeng X (2021). Artificial intelligence and optical coherence tomography for the automatic characterisation of human atherosclerotic plaques. EuroIntervention : Journal of EuroPCR in Collaboration with the Working Group on Interventional Cardiology of the European Society of Cardiology.

[36] Gao Z, Chung J, Abdelrazek M, Leung S, Hau WK, Xian Z (2020). Privileged Modality Distillation for Vessel Border Detection in Intracoronary Imaging. IEEE Transactions on Medical Imaging.

[37] Sano K, Kawasaki M, Ishihara Y, Okubo M, Tsuchiya K, Nishigaki K (2006). Assessment of vulnerable plaques causing acute coronary syndrome using integrated backscatter intravascular ultrasound. Journal of the American College of Cardiology.

[38] Stone GW, Maehara A, Lansky AJ, de Bruyne B, Cristea E, Mintz GS (2011). A prospective natural-history study of coronary atherosclerosis. The New England Journal of Medicine.

[39] Lawton JS, Tamis-Holland JE, Bangalore S, Bates ER, Beckie TM, Writing Committee Members (2022). 2021 ACC/AHA/SCAI Guideline for Coronary Artery Revascularization: A Report of the American College of Cardiology/American Heart Association Joint Committee on Clinical Practice Guidelines. Journal of the American College of Cardiology.

[40] Dangas GD, Claessen BE, Caixeta A, Sanidas EA, Mintz GS, Mehran R (2010). In-stent restenosis in the drug-eluting stent era. Journal of the American College of Cardiology.

[41] Min HS, Yoo JH, Kang SJ, Lee JG, Cho H, Lee PH (2020). Detection of optical coherence tomography-defined thin-cap fibroatheroma in the coronary artery using deep learning. EuroIntervention : Journal of EuroPCR in Collaboration with the Working Group on Interventional Cardiology of the European Society of Cardiology.

[42] Chu M, De Maria GL, Dai R, Benenati S, Yu W, Zhong J (2024). DCCAT: Dual-Coordinate Cross-Attention Transformer for thrombus segmentation on coronary OCT. Medical Image Analysis.

[43] Abdolmanafi A, Duong L, Ibrahim R, Dahdah N (2021). A deep learning-based model for characterization of atherosclerotic plaque in coronary arteries using optical coherence tomography images. Medical Physics.

[44] Zahnd G, Karanasos A, van Soest G, Regar E, Niessen W, Gijsen F (2015). Quantification of fibrous cap thickness in intracoronary optical coherence tomography with a contour segmentation method based on dynamic programming. International Journal of Computer Assisted Radiology and Surgery.

[45] Gessert N, Lutz M, Heyder M, Latus S, Leistner DM, Abdelwahed YS (2019). Automatic Plaque Detection in IVOCT Pullbacks Using Convolutional Neural Networks. IEEE Transactions on Medical Imaging.

[46] Liu R, Zhang Y, Zheng Y, Liu Y, Zhao Y, Yi L (2019). Automated Detection of Vulnerable Plaque for Intravascular Optical Coherence Tomography Images. Cardiovascular Engineering and Technology.

[47] Avital Y, Madar A, Arnon S, Koifman E (2021). Identification of coronary calcifications in optical coherence tomography imaging using deep learning. Scientific Reports.

[48] Gharaibeh Y, Prabhu D, Kolluru C, Lee J, Zimin V, Bezerra H (2019). Coronary calcification segmentation in intravascular OCT images using deep learning: application to calcification scoring. Journal of Medical Imaging (Bellingham, Wash.).

[49] Cho H, Kang SJ, Min HS, Lee JG, Kim WJ, Kang SH (2021). Intravascular ultrasound-based deep learning for plaque characterization in coronary artery disease. Atherosclerosis.

[50] Bae Y, Kang SJ, Kim G, Lee JG, Min HS, Cho H (2019). Prediction of coronary thin-cap fibroatheroma by intravascular ultrasound-based machine learning. Atherosclerosis.

[51] Masuda Y, Takeshita R, Tsujimoto A, Sahashi Y, Watanabe T, Fukuoka D (2025). Automated coronary artery segmentation / tissue characterization and detection of lipid-rich plaque: An integrated backscatter intravascular ultrasound study. International Journal of Cardiology.

[52] Mickley H, Veien KT, Gerke O, Lambrechtsen J, Rohold A, Steffensen FH (2022). Diagnostic and Clinical Value of FFR_CT_ in Stable Chest Pain Patients With Extensive Coronary Calcification: The FACC Study. JACC. Cardiovascular Imaging.

[53] de Oliveira Laterza Ribeiro M, Correia VM, Herling de Oliveira LL, Soares PR, Scudeler TL (2023). Evolving Diagnostic and Management Advances in Coronary Heart Disease. Life (Basel, Switzerland).

[54] Kang SJ, Ahn JM, Song H, Kim WJ, Lee JY, Park DW (2012). Usefulness of minimal luminal coronary area determined by intravascular ultrasound to predict functional significance in stable and unstable angina pectoris. The American Journal of Cardiology.

[55] Park SJ, Kang SJ, Ahn JM, Shim EB, Kim YT, Yun SC (2012). Visual-functional mismatch between coronary angiography and fractional flow reserve. JACC. Cardiovascular Interventions.

[56] Guo B, Jiang M, Guo X, Tang C, Zhong J, Lu M (2024). Diagnostic and prognostic performance of artificial intelligence-based fully-automated on-site CT-FFR in patients with CAD. Science Bulletin.

[57] Guo B, Xing W, Hu C, Zha Y, Yin X, He Y (2024). Clinical Effectiveness of Automated Coronary CT-derived Fractional Flow Reserve: A Chinese Randomized Controlled Trial. Radiology.

[58] Lee JG, Ko J, Hae H, Kang SJ, Kang DY, Lee PH (2020). Intravascular ultrasound-based machine learning for predicting fractional flow reserve in intermediate coronary artery lesions. Atherosclerosis.

[59] Cha JJ, Son TD, Ha J, Kim JS, Hong SJ, Ahn CM (2020). Optical coherence tomography-based machine learning for predicting fractional flow reserve in intermediate coronary stenosis: a feasibility study. Scientific Reports.

[60] Ali ZA, Maehara A, Généreux P, Shlofmitz RA, Fabbiocchi F, Nazif TM (2016). Optical coherence tomography compared with intravascular ultrasound and with angiography to guide coronary stent implantation (ILUMIEN III: OPTIMIZE PCI): a randomised controlled trial. Lancet (London, England).

[61] Flattery E, Rahim HM, Petrossian G, Shlofmitz E, Gkargkoulas F, Matsumura M (2020). Competency-Based Assessment of Interventional Cardiology Fellows' Abilities in Intracoronary Physiology and Imaging. Circulation. Cardiovascular Interventions.

[62] Mendizabal-Ruiz EG, Rivera M, Kakadiaris IA (2013). Segmentation of the luminal border in intravascular ultrasound B-mode images using a probabilistic approach. Medical Image Analysis.

[63] Matsumura M, Mintz GS, Dohi T, Li W, Shang A, Fall K (2023). Accuracy of IVUS-Based Machine Learning Segmentation Assessment of Coronary Artery Dimensions and Balloon Sizing. JACC. Advances.

[64] Galo J, Chaturvedi A, Al-Qaraghuli A, Rubio PM, Zhang C, Kahsay Y (2025). Machine Learning in Intravascular Ultrasound: Validating Automated Lesion Assessment for Complex Coronary Interventions. Catheterization and Cardiovascular Interventions : Official Journal of the Society for Cardiac Angiography & Interventions.

[65] Min HS, Ryu D, Kang SJ, Lee JG, Yoo JH, Cho H (2021). Prediction of Coronary Stent Underexpansion by Pre-Procedural Intravascular Ultrasound-Based Deep Learning. JACC. Cardiovascular Interventions.

[66] Kobayashi N, Mintz GS, Witzenbichler B, Metzger DC, Rinaldi MJ, Duffy PL (2016). Prevalence, Features, and Prognostic Importance of Edge Dissection After Drug-Eluting Stent Implantation: An ADAPT-DES Intravascular Ultrasound Substudy. Circulation. Cardiovascular Interventions.

[67] Chamié D, Bezerra HG, Attizzani GF, Yamamoto H, Kanaya T, Stefano GT (2013). Incidence, predictors, morphological characteristics, and clinical outcomes of stent edge dissections detected by optical coherence tomography. JACC. Cardiovascular Interventions.

[68] Maehara A, Matsumura M, Ali ZA, Mintz GS, Stone GW (2017). IVUS-Guided Versus OCT-Guided Coronary Stent Implantation: A Critical Appraisal. JACC. Cardiovascular Imaging.

[69] Leone AM, Rebuzzi AG, Burzotta F, De Maria GL, Gardi A, Basile E (2019). Stent malapposition, strut coverage and atherothrombotic prolapse after percutaneous coronary interventions in ST-segment elevation myocardial infarction. Journal of Cardiovascular Medicine (Hagerstown, Md.).

[70] Lombardi M, Chiabrando JG, Romagnoli E, D'Amario D, Leone AM, Aurigemma C (2023). Impact of acute and persistent stent malapposition after percutaneous coronary intervention on adverse cardiovascular outcomes. Minerva Cardiology and Angiology.

[71] Ong DS, Jang IK (2015). Causes, assessment, and treatment of stent thrombosis--intravascular imaging insights. Nature Reviews. Cardiology.

[72] Prati F, Kodama T, Romagnoli E, Gatto L, Di Vito L, Ramazzotti V (2015). Suboptimal stent deployment is associated with subacute stent thrombosis: optical coherence tomography insights from a multicenter matched study. From the CLI Foundation investigators: the CLI-THRO study. American Heart Journal.

[73] Nishi T, Yamashita R, Imura S, Tateishi K, Kitahara H, Kobayashi Y (2021). Deep learning-based intravascular ultrasound segmentation for the assessment of coronary artery disease. International Journal of Cardiology.

[74] Wissel T, Riedl KA, Schaefers K, Nickisch H, Brunner FJ, Schnellbaecher ND (2022). Cascaded learning in intravascular ultrasound: coronary stent delineation in manual pullbacks. Journal of Medical Imaging (Bellingham, Wash.).

[75] Bouma BE, Tearney GJ, Yabushita H, Shishkov M, Kauffman CR, DeJoseph Gauthier D (2003). Evaluation of intracoronary stenting by intravascular optical coherence tomography. Heart (British Cardiac Society).

[76] Zhao Wang, Jenkins MW, Linderman GC, Bezerra HG, Fujino Y, Costa MA (2015). 3-D Stent Detection in Intravascular OCT Using a Bayesian Network and Graph Search. IEEE Transactions on Medical Imaging.

[77] Xu C, Schmitt JM, Akasaka T, Kubo T, Huang K (2011). Automatic detection of stent struts with thick neointimal growth in intravascular optical coherence tomography image sequences. Physics in Medicine and Biology.

[78] Tsantis S, Kagadis GC, Katsanos K, Karnabatidis D, Bourantas G, Nikiforidis GC (2012). Automatic vessel lumen segmentation and stent strut detection in intravascular optical coherence tomography. Medical Physics.

[79] Lu H, Gargesha M, Wang Z, Chamie D, Attizzani GF, Kanaya T (2012). Automatic stent detection in intravascular OCT images using bagged decision trees. Biomedical Optics Express.

[80] Yang G, Mehanna E, Li C, Zhu H, He C, Lu F (2021). Stent detection with very thick tissue coverage in intravascular OCT. Biomedical Optics Express.

[81] Kumari V, Katiyar A, Bhagawati M, Maindarkar M, Gupta S, Paul S (2025). Transformer and Attention-Based Architectures for Segmentation of Coronary Arterial Walls in Intravascular Ultrasound: A Narrative Review. Diagnostics (Basel, Switzerland).

[82] Wang Q, Xu L, Wang L, Yang X, Sun Y, Yang B (2023). Automatic coronary artery segmentation of CCTA images using UNet with a local contextual transformer. Frontiers in Physiology.

[83] Shin D, Sami Z, Cannata M, Ciftcikal Y, Caron E, Thomas SV (2025). Artificial Intelligence in Intravascular Imaging for Percutaneous Coronary Interventions: A New Era of Precision. Journal of the Society for Cardiovascular Angiography & Interventions.

[84] Rubio PM, Garcia-Garcia HM, Galo J, Chaturvedi A, Case BC, Mintz GS (2026). Artificial intelligence-powered software outperforms interventional cardiologists in assessment of IVUS-based stent optimization. Cardiovascular Revascularization Medicine : Including Molecular Interventions.

[85] Sheikhtaheri A, Hasani N, Hosseini A (2023). Effect of picture archiving and communication system on diagnosis accuracy in CT and radiography examinations in emergency departments. International Journal of Medical Informatics.

